# Drug-Related Cutaneous Adverse Events in Philadelphia Chromosome-Negative Myeloproliferative Neoplasms: A Literature Review

**DOI:** 10.3390/ijms21113900

**Published:** 2020-05-30

**Authors:** Alessandra Malato, Elena Rossi, Giuseppe Alberto Palumbo, Paola Guglielmelli, Novella Pugliese

**Affiliations:** 1 UOC di Ematologia I ad indirizzo oncologico, Ospedali Riuniti Villa Sofia-Cervello, 90146 Palermo, Italy; 2 Dipartimento di Diagnostica per Immagini, Radioterapia Oncologica ed Ematologia, Fondazione Policlinico A. Gemelli IRCCS, 00168 Rome, Italy; elena.rossi@unicatt.it; 3 Sezione di Ematologia, Dipartimento di Scienze Radiologiche ed Ematologiche, Università Cattolica del Sacro Cuore, 00168 Rome, Italy; 4 Dipartimento di Scienze Mediche, Chirurgiche e Tecnologie Avanzate “G.F. Ingrassia”, University of Catania, 95123 Catania, Italy; giuseppealberto.palumbo@gmail.com; 5 CRIMM-Centro Ricerca e Innovazione delle Malattie Mieloproliferative, Department of Experimental and Clinical Medicine, Azienda ospedaliera-Universitaria Careggi, University of Florence, 50139 Florence, Italy; paola.guglielmelli@unifi.it; 6 Department of Clinical Medicine and Surgery, University of Naples Federico II, 80131 Naples, Italy; novypugliese@yahoo.it

**Keywords:** adverse events, Philadelphia chromosome-negative myeloproliferative neoplasms, cytoreductive agents

## Abstract

Since myeloproliferative neoplasms (MPN) pose a significant risk for vascular and thrombotic complications, cytoreductive therapies, such as hydroxyurea (HU), interferon (IFN) inhibitors, and Janus kinase (JAK) inhibitors are recommended for patients at high risk. However, these agents also place patients at increased risk for drug-related cutaneous adverse events. Herein, we review the literature on skin toxicity related to the use of drugs for the treatment of MPN. Overall, the cytoreductive agents used for MPN are generally well tolerated and considered to be safe, except IFN, for which dropout rates as high as 25% have been reported. While IFN is known to give rise to flu syndrome, it rarely leads to hematological alterations. The most common hematological side effects of HU are mild and include granulocytopenia, anemia, and thrombocytopenia. The JAK inhibitor ruxolitinib has been associated with cytopenia and a higher incidence of viral infections, as well as increased risk for basal cell carcinoma (BCC) and squamous cell carcinoma (SCC). Based on the present analysis, it can be concluded that cutaneous toxicity is not a negligible complication of commonly used treatments for MPN. While further research is needed, patients on these agents, and especially those with a history of cutaneous malignancies, should undergo thorough skin examination before and during therapy. In addition, detailed history is critical since many patients who develop non-melanoma skin cancer have multiple preexisting risk factors for cutaneous carcinogenesis.

## 1. Introduction

As myeloproliferative neoplasms (MPN) are characterized by elevated red blood cell mass, significant thrombocytosis, leukocytosis, and/or massive splenomegaly, they also pose a significant risk for development of both vascular and thrombotic complications. Conventional therapeutic options are important in order to minimize vascular and thrombotic risk; in this regard, for patients with a low risk of thrombotic events, first-line recommendations include daily low-dose aspirin and phlebotomy, while cytoreductive therapies, such as hydroxyurea (HU) or inhibitors of interferon (IFN) alpha or Janus kinase (JAK) are recommended for patients at high risk. One possible adverse event related to prophylaxis with HU is the development of mucocutaneous ulcers. These may occur at the beginning of therapy or at later times, even if the clinical presentation is similar [1]. While oral alterations are infrequent, they may, however, have significant clinical impact due to severe pain and impairment of feeding and speech. The available data appears to suggest that oral ulceration is one of the first HU-related adverse events in some patients, although it presents after a widely variable time after initiation of therapy, even after several years [2]. The physiological mechanisms at the basis of the skin and mucosal adverse effects due to HU remain unclear. Notwithstanding, a variety of cutaneous abnormalities have been reported during long-term therapy with HU, including xerosis, ichthyosis, pigmentation of nails, malleolar ulceration, and even malignant lesions.

The JAK family of intracellular, non-receptor tyrosine kinases have a role in signal transduction in many diseases. In this regard, the V617F mutation in JAK2, leading to gain in function, is a recurring feature of myeloproliferative disorders, and patients harboring this mutation have been shown to have a longer duration of disease and are also at increased risk for fibrosis. Ruxolitinib is a selective JAK1/JAK2 inhibitor used to treat MPN. In myelofibrosis (MF), ruxolitinib has been shown to improve 5-year overall survival vs. best available therapy with significant benefit in quality of life. However, basal cell carcinoma (BCC) or squamous cell carcinoma (SCC) developed in 17.1% of patients on ruxolitinib vs. 2.7% of those on the best available therapy [3]. Thus, it is likely that therapy with ruxolitinib may put some patients at greater risk of cutaneous malignant transformation, although the etiology of this rare side effect remains poorly understood. Herein, we conducted a literature review on the occurrence, side effects and possible etiology of cutaneous toxicities associated with ruxolitinib, HU, and interferon.

## 2. Ruxolitinib

As a selective JAK1/JAK2 inhibitor, ruxolitinib interferes with cytokine signaling and growth factors that play a role in hematopoiesis and immune function; JAK signaling also involves recruitment of signal transducers and activators of transcription (STATs) to cytokine receptors, thereby modulating the expression of several genes [4]. Ruxolitinib leads to a clinically significant reduction in spleen size and overall symptom burden in most patients with MF and seems to offer benefits in terms of survival. In polycythemia vera (PV), ruxolitinib has been shown to control hematocrit levels. Ruxolitinib is also being investigated as treatment for graft-versus-host disease (GvHD) following allogeneic hematopoietic stem cell transplantation (HSCT). In PV, ruxolitinib brings about rapid and lasting improvement in splenomegaly, improved control of symptoms and quality of life compared to best available therapy [4]. The detection of mutations in JAK2/MPL in patients with MPN paved the way for clinical development of JAK kinase inhibitors, such as ruxolitinib. In MF, ruxolitinib has been shown to improve splenomegaly, systemic symptoms, and overall survival; however, the precise mechanism by which JAK inhibitors achieve their efficacy remains unclear, even if several studies have indicated that the JAK/STAT pathway is important for signaling of various cytokines, which are related to regulation of inflammatory and immune responses.

The role of T-cell subsets in the skin and associated cytokines that signal through the JAK-STAT pathway is crucial for immune surveillance, considering that genetic defects in this signaling facilitate skin infection [5]. In this regard, inhibition of JAK-STAT signaling is an appealing therapeutic strategy in numerous immune-mediated dermatoses such as atopic dermatitis, alopecia areata (AA), psoriasis, and vitiligo [6,7]. 

At present, it is known that ruxolitinib modulates immune cell activities in several ways, including reduction of T regulatory cells (Tregs), silencing of T helper cells and a decrease of cytokine secretion [8,9]. Tregs are crucial for maintenance of self-tolerance and prevention of autoimmunity [10], and also have an important contribution in establishing cancer-induced immune escape in the skin [11,12]. Furthermore, many immunosuppressive agents can promote the development of cutaneous malignancies [11,13]. The type, levels, and duration of immunosuppressive therapy, as well as individual risk factors, influence the onset of skin cancer. In particular, the aggressive immunosuppressive therapy used for solid-organ transplantation has the consequence that skin cancer is the most common cancer in this setting [13].

Modifications in the skin immune microenvironment can be induced by ruxolitinib. Reports of aggressive skin cancer in patients treated with ruxolitinib suggest the need for appropriate surveillance of cutaneous malignancies. Commonly used immune-suppressive drugs, such as corticosteroids, cyclosporine, thiopurines, tacrolimus, mycophenolate mofetil, and sirolimus, all have intrinsic carcinogenic effects, in contrast to ruxolitinib which has no such intrinsic propriety [11]. It is possible that the lower incidence of skin cancer in patients treated with ruxolitinib compared to other immuno-modulating agents is due to the absence of a direct carcinogenic effect. Furthermore, after the first six months of ruxolitinib therapy, the number of T helper (Th)-17 cells is increased, which may represent an attempt at the reinstatement of immune surveillance against malignant MPN cells and skin malignancy [9].

### Cutaneous Adverse Events with Ruxolitinib

Adverse events related to ruxolitinib therapy include myelosuppression, leading to dose-limiting thrombocytopenia and anemia, as well as viral reactivation [14,15]. In addition, there are several reports of an increased rate of SCC and BCC (Table 1). 

Prospective trials in PV and MF have found that the increased rate of nonmelanoma skin cancers (NMSCs) with ruxolitinib is worthy of note [24,25,26,27]. The COMFORT and RESPONSE trials both excluded patients who had active malignancy within five years before enrolment, except for some skin malignancies. The trials reported that combined incidence of BCC and SCC were, respectively, 2.7 and 3.9 (COMFORT-1) and 6.1 and 3.0 (COMFORT-2) per 100 patient-years of exposure in the ruxolitinib and control groups. In RESPONSE, (4 and 2 patients in the ruxolitinib and standard-therapy arms had newly diagnosed BCC or SCC; all but one of these patients (in the standard-therapy group) had previous history of NMSC or suspicious skin lesions [27]. In the standard-therapy group, one patient was diagnosed with melanoma on day 155; the exposure-adjusted rate of NMSC in patients treated with ruxolitinib or standard therapy per 100 patient-years, respectively, was 5.1 (week 208) and 4.4 (week 80), compared to 2.6 (week 208) and 2.0 (week 80) in the group that crossed over. Moreover, these rates were higher in patients who had previous history of NMSC.

Fabiano et al. described eruptive SCC with keratoacanthoma-like features in a 74-year-old woman with MF who had been treated with ruxolitinib [21]. Chatterjee et al. described a 77-year-old patient with post-essential thrombocythemia MF who developed Sweet’s syndrome (a rare reactive skin condition characterized by fever, leukocytosis, and painful skin lesions) following treatment with ruxolitinib [18]. Blechman et al. published a small series of five cases of MF undergoing treatment with ruxolitinib who developed multiple aggressive skin cancers [17]. These authors suggested that the high rates of NMSCs might be related to the long duration of follow-up, especially considering the patient population. In this series, there was a case of lentigo maligna melanoma, while metastatic undifferentiated pleomorphic sarcoma was reported in another patient. Both of these cases, however, had several preexisting and significant risk factors for cutaneous cancer.

Dasanu presented an uncommon case of erythematous skin eruption with necrotic foci that involved the lower extremities in a patient with MF who was treated with ruxolitinib [19]. Loscocco et al. presented a case of Kaposi sarcoma (KS) in a patient who was enrolled in a phase 2 trial of ruxolitinib in PV or essential thrombocythemia (ET) that was refractory or intolerant to HU [23]. Ruxolitinib was discontinued by tapering over 2 weeks, after which progressive spontaneous regression of cutaneous lesions was achieved that largely resolved within 10 months.

Merkel cell carcinoma (MCC) is an aggressive, rare neuroendocrine carcinoma and a common cause of skin cancer-related death. Of note, a case of MCC has been reported following treatment with ruxolitinib for PV [28]. This is likely related to the fact that the immune system plays a fundamental role in preventing MCC and in inhibiting its progression, and about one-tenth of patients with MCC are immune suppressed. Administration of ruxolitinib to patients reduces both splenomegaly and levels of proinflammatory cytokines, and also helps to clear neoplastic cells harboring a mutated JAK2 gene. Moreover, inhibitors of JAK1/2 reduce the activity of selected cytokines such as IL-6 and IL-23, thereby inhibiting the production of several other proinflammatory cytokines, chemokines, and adhesion molecules, leading to interruption of the cytokine signaling cascade [8,29]. 

The reports of aggressive skin cancers following therapy with a JAK inhibitor thus suggest that these agents may favor malignant transformation in the skin in patients who are at high risk. Accordingly, watchful surveillance regarding the risk of skin cancer is strongly warranted.

Among the six case reports of skin toxicity in ruxolitinib-treated patients in whom previous therapies were described, three had been previously treated with HU. One can thus speculate that previous therapy may be associated with increased risk of a secondary skin adverse event. Although further research is warranted on the potential role of JAK inhibitors such as ruxolitinib in the development of skin lesions, patients on therapy with a JAK inhibitor should undergo routine skin examination, and especially individuals who have a history of skin cancer. Specialists such as dermatologists should also be aware of this possibility so that appropriate preventive strategies can be implemented.

## 3. Hydroxyurea

HU is a non-alkylating agent that is widely employed for treatment of chronic MPNs. HU exhibits non-competitive inhibition of ribonucleotide reductase, which leads to the depletion of deoxyribonucleotides. As a result, DNA synthesis is interrupted and the cell cycle blocked in S-phase [30]. As ribonucleotide reductase is involved in DNA repair, HU also induces double-stranded breaks in DNA [30]. Although HU is generally well tolerated, its widespread use, not only in MPNs, has revealed the presence of adverse events related to tissues that have a high cellular turnover due to the cytostatic action of HU. Multiple cutaneous alterations have been described in patients treated with HU. Among these, cutaneous ulcers and non-melanoma skin cancer lead to treatment discontinuation due to inacceptable toxicity with consequent modification of the treatment approach.

### Cutaneous Adverse Events Associated with Hydroxyurea

Cutaneous ulcers, typically in the perimalleolar area, are probably an underestimated side effect of HU. The pathogenesis of these lesions is likely multifactorial, and the cytotoxic effects of HU on basal cells of the epidermidis, keratinocytes, and endothelial cells likely plays a key role. HU-induced macrocytosis has also been recognized as a possible cause of microvascular disturbance due to deformability of red blood cells in capillaries and reduced oxygenation of the basal layer of the skin [31]. On the other hand, MPNs induce alterations in both arterial and venous circulation, likely contributing to ischemia and delays in wound repair [31]. However, cutaneous ulcers may often occur after long-term treatment with HU and when patients show hematological response.

Non-melanoma skin cancer (NMSC) and actinic keratosis (AK) have been reported to be induced by HU. Impaired DNA repair upon exposure to HU leads to somatic mutations and chromosomal damage, especially in sun-exposed areas, and UV-induced breaks in double-stranded DNA may also contribute to HU-mediated carcinogenesis.

**Figure 1:**
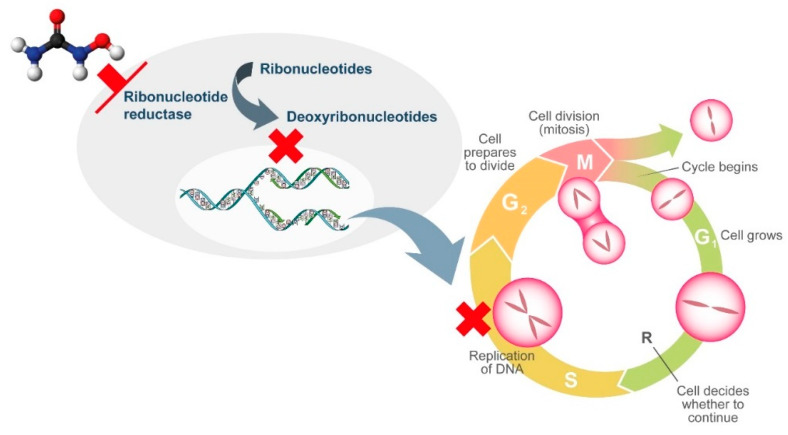
Mechanism of action of hydroxyurea.

**Figure 2:**
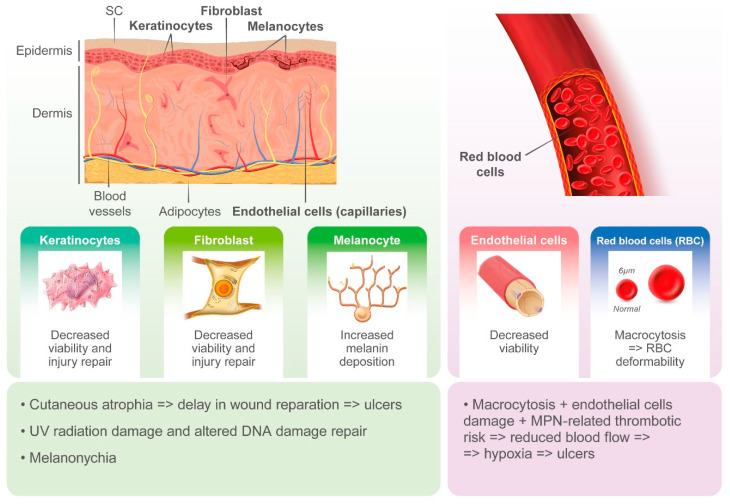
Pathogenesis of hydroxyurea-induced cutaneous toxicity.

Other HU-induced skin toxicities have only aesthetic implications, such as hyperpigmentation of skin and nails. Melanin deposition by melanocyte stimulation in the nail matrix by HU and photosensitization have been hypothesized as pathogenic mechanisms. Although alopecia represents a frequently described dermatological side effect with the use of more potent cytostatic drugs, it has also been reported in patients treated with HU. Its pathogenesis is related to the alterations in cell kinetics of the hair matrix HU (Figure 1 and Figure 2).

Ulcers are the most frequent reported cutaneous adverse event in patients with MPNs undergoing treatment with HU (Table 2). 

**Figure 3:**
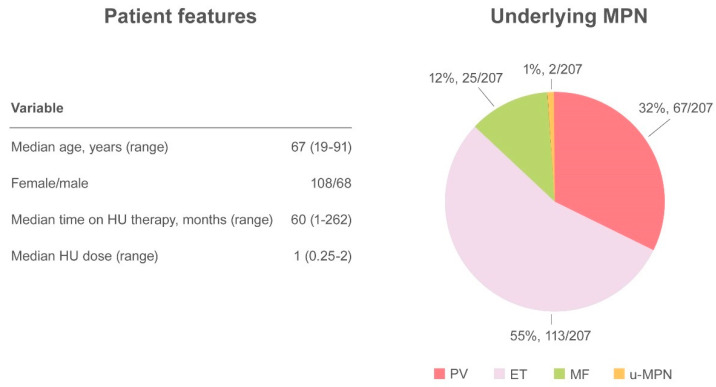
Summary of cutaneous ulcers as an adverse event.

Altogether, 27 papers have been published, including a randomized controlled clinical trial, retrospective studies, case series, and case reports, accounting for a total of 249 cases [33,34,38,39,40,44,51,52,53,54,55,56,57,58,59,60,61,62]. The demographic and clinical features of MPN have not always have been described, but when available the median age of patients with skin ulcers was 67 years (range 19–91) and there was a higher prevalence of HU-related ulcers in women (61.4%, 108/176) compared with men (38.6%, 68/176). Underlying MPN pathologies were distributed as follows: PV 32.4% (67/207), ET 54.6% (113/207), MF 12.1% (25/207), and u-MPN 1% (2/207). The median time from initiation of HU to the detection of a skin ulcer was 60 months (range 1–262) at a median daily dose of 1 g (range 0.25–2) [33,34,38,39,40,44,45,51,52,53,54,55,56,57,58,59,60,61,62,63,64,65,66]. The most frequent localization was the leg, at the perimalleolar side, and concomitant venous or arterial insufficiency was reported in 38 cases. To ensure healing of the lesion, discontinuation of therapy is mandatory and was described in virtually all cases in which intervention was specified (Figure 3).

**Figure 4:**
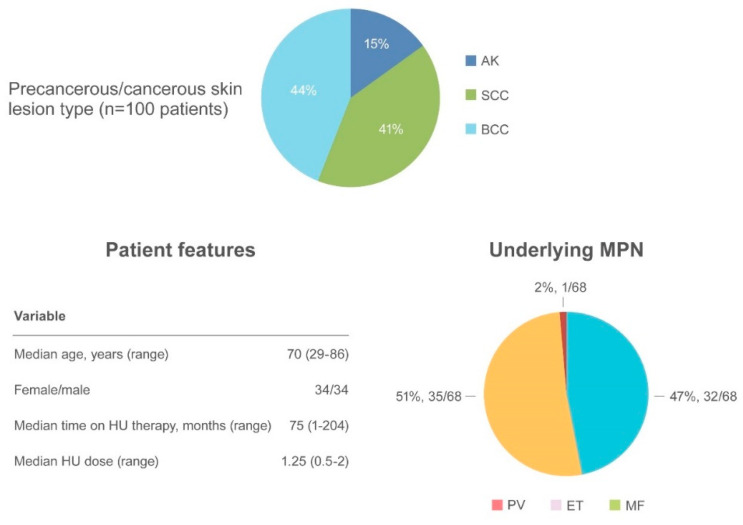
Summary of actinic keratosis and non-melanoma skin cancer as an adverse event.

AK and NMSC are notable adverse events in HU-treated patients (Figure 4). 

AK has been reported in 15 patients [57,63,67,68,69,70,71] often preceding the appearance of NMSC. SCC occurred in 41 patients and BCC in 44 patients [32,44,52,57,59,62,63,69,70,72,73]. The median age of onset was 70.6 years (range 29–86), with a similar incidence of NMSC in women (34 patients) and men (34 patients). The most frequently involved regions were photo-exposed areas, such as the scalp (43 patients), ears/neck (5 patients), hands (5 patients), and diffuse pattern (5 patients). When reported, the underlying MPN disease were as follows: PV (32/68, 47%), ET (35/68, 51.5%) and MF (1/68, 1.5%). The median time to NMSC after initiating HU was 75 months (range 1–204) at a median HU daily dose of 1.25 g (range 0.5–2). Surgical excision of the suspected lesion and HU discontinuation were the most frequent types of intervention. 

## 4. Interferons

In almost all trials recruiting patients with MPNs, IFN-α therapy induced a rapid hematological response (Table 3). It is noted that there is wide variability in the underlying disease, type of interferon, dose, timing, and duration of treatment among the different trials. In most trials, it is not stated when/if the IFN was discontinued. However, IFN-α led to discontinuation of treatment due to toxicity in nearly one-fourth of patients among those reporting discontinuations.

### Cutaneous Adverse Events Associated with Interferons

Cutaneous adverse events only rarely required interruption of IFN. Local injection site reactions (ISR), such as tenderness with or without symptoms such as warmth, erythema, itching, less frequently lipodystrophy, edema, and phlebitis, have been reported in 33 patients, independently of the underlying disease, and the type, dose, and duration of IFN. The ISR was complicated by infection in only a single case. While such symptoms usually appear within a short timeframe after initiating IFN, they may also appear years later. However, a realistic evaluation of ISR is not possible since patients may not report events due to self-injection or mild grade, which rarely lead to discontinuation.

**Figure 5:**
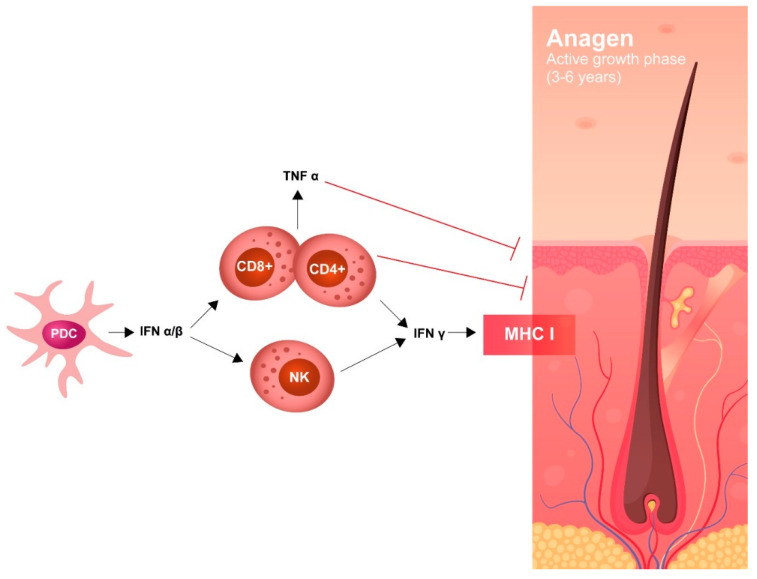
Pathogenesis of IFN-induced alopecia.

AA has been described in 42 patients treated with any formulation of IFN-α2, both pegylated and non-pegylated, including pegylated IFN-α2b (ropeginterferon). The underlying disease (ET, PV, MF), IFN dose, schedule, and treatment duration do not seem to influence the appearance of AA. Autoimmune assault of anagen hair follicles by both the innate and adaptive immune system causes non-scarring hair loss; IFNs may have a role in this process, as there are several reports of induction/exacerbation of AA following treatment with an IFN. The severity of hair loss ranges widely and most cases are not well described (Figure 5).

Recent studies have suggested that high levels of IFN-γ in lesions related to alopecia induces several chemokines (CXCL9, CXCL10) which amplify a T cell response around hair bulbs, causing targeting of specific autoantigens that are responsible for the loss of hair. In AA, JAK-STAT signaling is recognized to be an important pathway; in this regard, and several recent trials with JAK inhibitors in AA have shown promising results.

Tichelli et al. described 10 cases of dry scaly skin in 13 patients treated with IFN-α2a for MPN; in two patients, extended erythematous plaques were also present [83]. Besides, Langer et al. reported the appearance of dry skin in 10 patients among 36 ET patients treated with PEG-α2b at a weekly dose of 50 mcg [78]. Among diffuse skin side effects, three patients developed skin rash/erythema and seven experienced itching, which were not related to MPN.

## 5. Relative Risk of Different Agents

**Figure 6:**
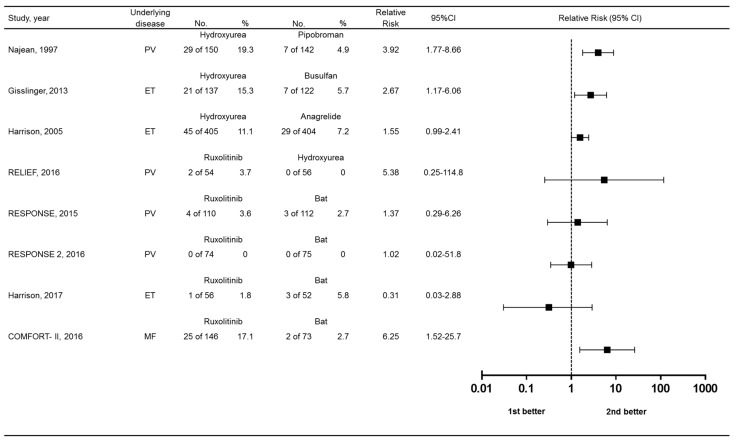
Forest plot showing relative risk of skin-related drug-related adverse events. Bat, best available therapy.

We lastly compared the relative risks of skin-related drug-related adverse events among randomized controlled trials comparing MPN treatments. Risk ratios with 95% confidence intervals (CIs) and are in Figure 6. Compared to best available therapy, only the COMFORT-II trial showed an advantage for ruxolitinib. Hydroxyurea appeared to be associated with greater risk over the comparator used in clinical trials, except for the RELIEF study vs. ruxolitinib.

## 6. Discussion

The cytoreductive agents employed in MPN are usually well-tolerated and considered safe, with the exception of IFN for which dropout rates as high as 25% have been reported [83]. IFN is known to give rise to flu syndrome, and rarely to hematological alterations such as thrombocytopenia or liver toxicity. The most common hematological side effects of HU are mild and include granulocytopenia, anemia, and thrombocytopenia. Only a few cases of fever and constipation, or more rarely diarrhea, have been reported. Ruxolitinib, the most recent addition to the therapeutic armamentarium for MPN, may lead to cytopenia, especially anemia and thrombocytopenia, as well as a higher incidence of viral infections.

In this review, we focused on the cutaneous toxicities reported during administration of these three drugs in patients with MPN. IFNs are associated with ISRs, but are only rarely a cause of discontinuation. However, evaluation of this side effect is not easy, given that potential mistakes in injection procedures and mild events are not reported during self-administration. Skin dryness with eventual erythematous complications is responsible for pruritus that is not MPN-related, which sometimes appears in patients when treated with IFNs [78]. AA has been described with the use of IFNs and the autoimmune mechanisms implicated in the pathogenesis of this disease are likely amplified by IFNs, which may explain the report of AA during the use of IFNs.

Loss of immunosurveillance, especially because of the effect on subsets of T cells, may help to explain the increase in non-melanoma skin neoplasms in patients treated with ruxolitinib [8]. The action of ruxolitinib, a selective JAK inhibitor, is due to interference with JAK-STAT signaling pathway. Inhibition of cytokines and growth factors in this pathway is effective in controlling hematopoiesis and immune functions. The specific target of the drug can also explain the observed cutaneous effects: it is known that iatrogenic immunosuppression due to pharmacological treatments may be a major cause of non-melanoma skin cancer. The exact role of ruxolitinib in modulation of immune cells is not completely clear at present; it does not appear to have a direct cancerogenic effect, but rather provokes modification in the skin immune microenvironment. It is important to note that in COMFORT and RESPONSE trials, where the incidence of cutaneous BCC and SCC were higher than in the control arm, patients with a previous history of skin cancer or precancerous skin lesions were not excluded and the majority of patients who developed NMSC during treatment with ruxolitinib had positive history [24,25,26,27].

The mechanism of action of HU is less associated with immune control. HU acts as antineoplastic agent by interfering with DNA synthesis, thus blocking the cell cycle. Unlike IFNs and ruxolitinib, the direct effect of HU on tissues with high cellular turnover, such as the skin, are more intuitive [30]. The cytotoxic effects on epidermal cells, keratinocytes and endothelial cells, HU-induced microvascular disturbances due to macrocytosis, and compromised DNA repair following UV damage are the main pathogenetic ways [31]. The occurrence of painful leg ulcers has been described during treatment with HU [91]. Their incidence may be underestimated, especially in patients receiving higher doses and after long term use [31]; in such cases, discontinuation and switching to alternative therapies can be recommended [92].

Other mild mucocutaneous alterations, such as hyperpigmentation in nails, AA, and scaling have been reported with HU, in addition to severe toxicities (oral aphthosis, dermatomyositis-like eruptions) [51]. Other than leg ulcers, an exhaustive description of these skin toxicities is still lacking. With regards to secondary neoplasms, HU was associated with a risk of NMSC that was two-fold higher (OR 2.28; 95% CI 1.15–4.51) [93].

## 7. Materials and Methods

Case reports and clinical studies were reviewed to summarize the literature on skin toxicity related to the use of drugs for treatment of MPN. A search of the MEDLINE and TOXLINE databases retrieved 82 articles: 74 case reports, 5 retrospective studies, and 3 prospective studies. Data were extracted for the following: medical anamnesis (gender, age, comorbidities; presence of venous or arterial insufficiency, diabetes, hypertension, previous skin trauma at the site of lesion, cutaneous abnormalities related to HU, interferon, ruxolitinib); pharmacological history (duration, daily and cumulative dose of HU, use of IFN or other drugs); characteristics of ulcers (site, multiple or bilateral lesions, duration); therapy for MPN and skin lesion (HU, hematologic therapy, time of healing, final outcome). Randomized controlled trials comparing MPN treatments that estimate drug-related skin adverse event incidence were identified from electronic databases. Outcome variables are shown as risk ratios (RRs) with 95% confidence intervals (CIs) and were compared by qualitative and quantitative syntheses (meta-analyses).

## 8. Conclusions

From our analysis, we conclude that cutaneous toxicity is not a negligible complication of commonly used treatments for MPN. Although further research is needed, and especially in better understanding of the pathophysiological basis of skin lesions in MPN patients, we suggest that patients on these medications, and especially those with a history of cutaneous malignancies, should undergo accurate skin examination before starting therapy followed by routine examinations during therapy. Obtaining a detailed anamnesis is fundamental given that many patients who develop NMSC have multiple preexisting risk factors for cutaneous carcinogenesis.

## Figures and Tables

**Table 1:** Reported cases of ruxolitinib-induced cutaneous toxicity.

Study	Study Type	Reported Case	Sex	Age	Underlying Disease	Driver Mutation Gene	Ruxolitinib Dose (mg/BID)	Duration of Treatment (months)	Toxicity Type	Site	Biopsy Performed	Ruxolitinib Discontinued	Intervention Type	Previous HU Therapy	Sun Exposure
**Aboul-Fettouh, 2018** [16]	Case report	1	F	70	PPV-MF	JAK2	10	60	SCC + BCC	Head and neck	Yes	Yes	Surgical excision	Yes	Yes
**Blechman, 2017** [17]	Case series	5	M	*60 (50–73)	PV	JAK2	*20 (5–25)	*28 (18–50)	5 SCC, 2 BCC, 1 UPS, 1 LMM	Diffuse	Yes	Yes	Surgical excision/radiotherapy/chemotherapy	Yes	*n/r*
**Chatterjee, 2015** [18]	Case report	1	F	77	PET-MF	*n/r*	*n/r*	18	Sweet syndrome	Diffuse	Yes	Yes	Oral Steroid	*n/r*	*n/r*
**Dasanu, 2018** [19]	Case report	1	M	73	PMF	JAK2	20	2	Erythematous skin lesion	Knee	Yes	No	Topical steroid	No	No
**Del Rosario, 2015** [20]	Case report	1	M	79	PMF	JAK2	15	7	Ulcer	Leg	Yes	No	Cephalexin	No	*n/r*
**Fabiano, 2015** [21]	Case report	1	F	74	PMF	*n/r*	*n/r*	2	SCC-keratoacanthoma type	Head and neck	Yes	Yes	Surgical excision	No	Yes
**Fournier, 2011** [22]	Case report	1	M	61	PPV-MF	*n/r*	20	1	Morbilliform lesion (Interstitial granulomatous drug reaction)	Diffuse	Yes	Yes	Topical steroid	*n/r*	*n/r*
**Loscocco, 2017** [23]	Case report	1	M	56	ET	CALR	*n/r*	84	BCC + Kaposi Sarcoma	Diffuse	Yes	Yes	Spontaneous regression	Yes	*n/r*

BID: Twice a day; ET: essential thrombocythemia; SCC: Squamous cell carcinoma; BCC: Basal cell carcinoma; UPS: undifferentiated pleomorphic sarcoma; LMM: lentigo malignant melanoma; PET-MF: post–essential thrombocythemia myelofibrosis; PMF: primary myelofibrosis; PPV-MF: post–polycythemia vera myelofibrosis; PV: polycythemia vera; n/r: not reported. * Data are reported as median and range.

**Table 2:** Reported cases of hydroxyurea-induced cutaneous toxicity.

Study	Study Type	Reported Case	Sex	Age	Underlying Disease	Driver Mutation Gene	HU Dose (g/daily)	Duration of Treatment (months)	Toxicity Type	Site	Biopsy Performed	HU Discontinued	Intervention Type	Vascular Insufficiency	Sun Exposure
**Antar, 2014** [32]	Case report	1	F	60	ET	JAK2	n/r	60	SSC	Leg	Yes	Yes	Surgical excision	n/r	n/r
**Bader, 2000** [33]	Case series	3	1 F, 3 M	84.6	2 PV, 1ET,	*n/r*	0.66 (0.5–1)	18–96	Ulcers	Leg	Yes (2)	Yes (2)	Oral steroid and skin split graft (1)	3	*n/r*
**Best, 1998** [34]	Case series	10	5 F, 4 M	64.1	5 PV, 2ET, 2 MF, 1 u-MPN	*n/r*	1.5 (1–2)	84 (3–15)	Ulcers	Diffuse	Yes	*n/r*	*n/r*	*n/r*	*n/r*
**Butler, 2014** [35]	Case report	1	M	64	PV	JAK2	1.5	36	Acral erythema	Hand/foot	No	*n/r*	*n/r*	*n/r*	*n/r*
**Callot-Mellot, 1996** [36]	Case series	5	3 F, 2 M	71 (64–76)	2 PV, 3 ET	*n/r*	*n/r*	78 (24–120)	2 SCC, 3 BCC, actinic keratosis (5)	n/r	Yes	Yes	*n/r*	*n/r*	*n/r*
**Cohen, 1999** [37]	Case report	1	F	70	PV	*n/r*	2–4	48	Melanonychia	Fingernails and toenails	No	Yes	*n/r*	*n/r*	*n/r*
**Daoud, 1997** [38]	Case series	3	*n/v*	56-69	1 PV, 2 ET	*n/r*	*n/v*	61 (55–79)	3 ulcers, 1 poikilodermatous eruption	Palms, toes, dorsal feet, ankles	Yes	Yes	*n/r*	*n/r*	*n/r*
**De Benedettis, 2004** [39]	Case report	1	M	66	PV	*n/r*	1	204	Ulcers, SCC	Leg, oral SCC	Yes	Yes	Surgical excision	*n/r*	*n/r*
**Demicray, 2002** [40]	Case series	3	3 F	61.6 (56–65)	3 ET	*n/r*	1	50 (6–84)	Ulcers	Leg	Yes (2)	Yes (1/3)	Oral steroids	2/3	*n/r*
**Esteve, 2001** [41]	Case report	1	F	83	PV	*n/r*	*n/r*	156	Actinic keratosis, SCC	Hands	Yes	Yes	Surgical excision	*n/r*	*n/r*
**Hernandez-Martin, 1999** [42]	Case report	1	M	78	ET	*n/r*	1	5	Melanonychia	Fingernails and toenails	No	No	None	*n/r*	*n/r*
**Hirri, 2001** [43]	Case Report	1	M	66	u-MPN	*n/r*	1.5	8	Ulcers	Leg	No	Yes	None	*n/r*	*n/r*
**Hoff, 2009** [44]	Case report	1	F	68	PV	*n/r*	*n/r*	96	Ulcers, actinic keratosis, SCC	Leg, head	Yes	Yes	Surgical excision, cryotherapy	No	*n/r*
**Hwang, 2009** [45]	Case report	1	M	75	ET	*n/r*	2	48	Ulcers, melanonychia	Leg, fingernails and toenails	Yes	Yes	None	*n/r*	n/r
**Kelly, 1994** [46]	Case report	1	M	61	PV	*n/r*	1.5–2	72	Actinic keratosis, BCC	Diffuse	Yes	No	Surgical excision, topical steroids	*n/r*	Yes
**Kluger, 2011** [47]	Case report	1	F	74	ET	*n/r*	0.6	36	Melanonychia	Toenails	No	No	None	*n/r*	*n/r*
**Kwong, 1996** [48]	Case report	1	F	69	ET	*n/r*	2–3	6	Melanonychia	Fingernails and Toenails	No	n/r	None	*n/r*	*n/r*
**Simeonovski, 2018** [49]	Case report	1	M	52	ET	*n/r*	1.5	>120	Perimalleolar and nummular lesions, actinic keratosis, BCC	Less, arms, nose	Yes	Yes	Surgical excision, cryotherapy	*n/r*	*n/r*
**Accurso, 2019** [50]	Case report	1	F	72	MPN	JAK2	*n/r*	≈84	Desquamative dermatitis	Diffuse facial	No	Yes	Topical and systemic steroids	*n/r*	*n/r*

ET: Essential Thrombocythemia; MPN: Myeloproliferative neoplasms; PV: Polycythemia Vera; SSC: Squamous Cell Carcinoma; BCC: Basal Cell carcinoma; n/r: not reported.

**Table 3:** Reported cases of interferon (IFN)-induced cutaneous toxicity.

Study	No. of Patients	Sex	Age	Underlying Disease	IFN Type	IFN Dose	Duration of Treatment (Months)	Reported Cases	Type of Toxicity	Site	IFN Discontinued
**Seewann, 1991** [74]	36	18 F, 18 M	60 (26–73)	19 ET, 6 PV, 6 CML, 5 CMGM	α2b	5-3 MU/daily	n/r	17	14 Alopecia, 3 pruritus	*n/r*	*n/r*
**Kasparu, 1992** [75]	14	8 F, 6 M	65 (36–65)	14 ET	α2b	5 MU/daily		1	alopecia	*n/r*	*n/r*
**Radin, 2003** [76]	60	33 F, 27 M		17 ET, 12 PV, 31 MF	n/r	5-2 MU/daily	6	20	*n/r*	*n/r*	*n/r*
**Alvarado, 2003** [77]	11	9 F, 2 M	55 (26–69)	11 ET	PEG-α2b	4.5 mg/kg/week	25 (0–84)	8	5 ISR, 3 alopecia		*n/r*
**Langer, 2005** [78]	36	20 F/16 M	54 (24–72)	ET	PEG-α2b	50 mcg/weekly	23 (3–39)	20	7 hair loss, 13 skin dryness	Diffuse	2 females for alopecia
**Sammuelsson, 2006** [79]	42	20 F, 22 M	53 (29–77)	21 ET, 21 PV	PEG-α2b	0.5 mcg/kg	24	36	27 ISR1, 8 alopecia, 1 erythema		1 alopecia, 1 erythema
**Jabbour, 2007** [80]	40	n/r	54 (28–81)	13 ET, 4 PV, 11 PMF, 10 others	PEG-α2b	2–3 mcg/kg weekly	27 (4–42)	8	n/r	*n/r*	*n/r*
**Ludwing, 1987** [81]	15	11 F, 4 M	66 (54–80)	5 ET, 7 PV, 3 CML	α2c	5–10 MU/3-7 times a week	2	3	3 alopecia		*n/r*
**Abegg-Werter, 1990** [82]	8	5 F, 3 M	42 (29–63)	8 PV	α2c	0.5 mg/weekly	*n/r*	3	1 alopecia, 2itching		*n/r*
**Tichelli, 1989** [83]	13	6 F, 7 M	57(21–78)	3 ET, 4 PV, 6 others	α2a	9 MU/daily	*n/r*		10 dry scaly skin, 2 extended erythematous plaques, 4 alopecia, 1 ISR		*n/r*
**Bentley, 1999** [84]	34		41 (14–68)	ET	α2a	3 MU/daily	24	7	2 alopecia, 2 skin rash, 2 pruritus, 1 infected injection site		*n/r*
**Saba, 2005** [85]	23	14 F, 9 M	41 (20–63)	23 ET	α2a	5 MU/m^2^/daily	174 (9–202)		Alopecia (number not reported)		*n/r*
**Gilbert, 1998** [86]	54	21 F, 33 M	18–85	3 ET, 8 PV, 14 MF, 25 Smf, 4 Umpn	α2b	5 MU/daily	30 (1–97)		>5% Alopecia, ISR		*n/r*
**Kiladjan, 2008** [87]	37	21 F, 16 M	49 (42–53)	37 PV	PEG-α2a	90–135 mcg/weekly	31.4 (26.4–35.1)	6	*n/r*	*n/r*	1
**Silver, 2006** [88]	61	27 F, 28 M	51 (24–80)	61 PV	α2b/α2a	3 MU/m^2^/3 week		1	1 skin rash	*n/r*	*n/r*
**Heis, 1999** [89]	32	17 F, 15 M	60.5 (31/81)	32 PV	ALPHA	*n/r*	14 (2–126)	1	1 alopecia		*n/r*
**Gisslinger, 2015** [90]	51	20 F, 31 M	56 (35-82)	51 PV	Ropegalfa-2b	*n/r*	*n/r*	>10%	>10% Alopecia, ISR	Alopecia, ISR	
**Total**	567							131			

CML: Chronic Myeloid Leukemia; CMGM: Chronic megakaryocytic Granulocytic Myelosis; ET: Essential thrombocythemia; PV: Polycythemia vera; MF: Myelofibrosis; ISR: Injection site reactions; n/r: Not reported.

## Abbreviations

AA	Alopecia areata
AK	Actinic keratosis
BCC	Basal cell carcinoma
BID	Twice a day
ET	Essential thrombocythemia
HU	Hydroxyurea
IFN	Interferon
ISR	Injection site reactions
JAK	Janus kinase
KS	Kaposi sarcoma
MCC	Merkel cell carcinoma
MF	Myelofibrosis
MPN	Myeloproliferative neoplasms
NMSC	Nonmelanoma skin cancer
PET-MF	Post–essential thrombocythemia myelofibrosis
PMF	Primary myelofibrosis;
PPV-MF	Post–polycythemia vera myelofibrosis;
PV	Polycythemia vera
SCC	Squamous cell carcinoma
STATs	Signal transducers and activators of transcription
Th	T helper
Treg	T regulatory cell
